# The cancellation heuristic in intertemporal choice shifts people’s time preferences

**DOI:** 10.1038/s41598-022-07906-w

**Published:** 2022-03-17

**Authors:** Arjun Sengupta, Krishna Savani

**Affiliations:** 1 Alfred Weber Institute of Economics, Heidelberg University, Heidelberg, Germany; 2 Nanyang Business School, Nanyang Technological University, Singapore, Singapore; 3 The Hong Kong Polytechnic University, Hung Hom, Hong Kong; 4 Industrial Economics Department, University of Nottingham, Nottingham, UK

**Keywords:** Psychology, Human behaviour

## Abstract

Building on past research in risky decision making, the present research investigated whether the *cancellation heuristic* is evident in intertemporal choice. Specifically, the cancellation heuristic posits that whenever choice options are partitioned into multiple components, people ignore seemingly identical components and compare the non-identical components. We nudged people to employ the cancellation heuristic by partitioning both the smaller earlier reward and the larger later reward into a seemingly identical component and a non-identical component. Given diminishing marginal utility, we hypothesized that people would perceive an identical difference between the smaller earlier reward and the larger later reward as being subjectively greater when both amounts are smaller in magnitude, thereby increasing the relative attractiveness of the larger later reward in the partition condition. We conducted four studies, including two incentive-compatible lab experiments, one incentive-compatible lab-in-the-field experiment, and one survey study using choices among both gains and losses. We consistently found that this choice architecture intervention significantly increased people’s likelihood of choosing the larger later reward. Furthermore, we provide evidence of the underlying mechanism-people’s intertemporal decisions shifted to a greater extent in the cancellation condition, particularly if their marginal utility diminished faster. The findings indicate that two features of human psychology-diminishing marginal utility and the cancellation heuristic-can be simultaneously utilized to nudge people to make decisions that would be better for them in the long run.

## Introduction

Intertemporal impatience has been cited as one of the major shortcomings of human decision-making, with profound consequences for people’s health, wealth, and happiness^1–3^. In the present research, we investigate whether there exists a general decision-making heuristic-the cancellation heuristic-that can help people make more patient choices. Specifically, we propose that a choice architecture intervention (i.e., separating a seemingly identical amount from multiple options) would induce people to ignore the seemingly identical amount and just compare the residual amount. We further propose that this intervention can nudge people to make more patient choices when combined with a concave utility function. Partitioning options into cancellable components thus represents another choice architecture tool that policymakers can use to nudge individuals to make more patient decisions^4,5^.

To illustrate our proposed effect, imagine that you have just turned 62 and are eligible to receive US Social Security benefits. You face an intertemporal choice between receiving $7000 annually in Social Security benefits starting age 62 versus $10,000 annually starting five years later (at age 67, your full retirement age). Now consider the choice between receiving $6000 + $1000 starting this year and receiving $6000 + $4000 starting five years later. Although the two choices are identical, in the second choice, a common amount ($6000) is separated from both the smaller earlier and the larger later rewards. In the present research, we ask whether people adopt a general cancellation heuristic, whereby they mentally cancel out or ignore seemingly identical amounts across the different rewards, thereby reducing the options to receiving $1000 starting this year versus receiving $4000 starting five years later. Given diminishing marginal utility of wealth^6^, people are likely to perceive the difference between $1000 versus $4000 as bigger than the difference between $7000 versus $10,000. Therefore, we predicted that if a general cancellation heuristic exists, then separating an amount from both rewards would shift people’s preferences from the smaller earlier reward to the larger later reward.

The proposed cancellation heuristic in intertemporal choice has been previously documented in risky decision-making^7^. Specifically, when making risky decisions, people tend to ignore or cancel out identical elements of lotteries, which can lead to preference reversals^7^. This cancellation heuristic is related to the isolation effect in risky decision-making^8^. The isolation effect refers to people’s tendency to ignore common probabilities when making risky choices if those common probabilities are separated at a prior stage. Research has also demonstrated the isolation effect with respect to the time dimension of intertemporal choice using a two-stage procedure. When a common delay was isolated from both the smaller earlier and the larger later rewards, 22% more participants chose the smaller earlier reward over the larger later reward, indicating that many participants ignored the common delay and made choices as if one reward was immediate and the other delayed^9^.

The cancellation of common elements in risky choice^7^, the isolation of common delays in intertemporal choice^9^, and the isolation of common probabilities in risky choice^8^ are examples in which people ignore identical elements across options. Going beyond this work, the current research seeks to make three contributions to the intertemporal choice literature. First, using incentivized experiments, we ask whether representing choices with a common payoff separated (e.g., baseline: $3 today versus $7 in a week, cancellation: $3 + $0 today versus $3 +$4 in a week) substantially increases the proportion of people choosing the larger later option. Further, in the loss domain, people tend to prefer larger later losses; here, we test whether separating a common payoff from losses leads people to choose the smaller earlier loss more often. Thus, in both the gain and the loss domain, the cancellation nudge can lead people to more rational decisions.

Second, we investigate the interaction between the cancellation heuristic and the shape of people’s utility curve. Specifically, we test whether people with higher curvature utility function over wealth would be more susceptible to the cancellation heuristic. If people use the cancellation heuristic, they end up comparing two smaller rewards differing by the same amount as the two larger rewards. As diminishing marginal utility implies that an identical increase in payoff yields higher marginal utility if all amounts are smaller^6,8^, we hypothesized that more people would choose the larger later reward in the cancellation condition than in the control condition. Moreover, our argument predicts that the cancellation framing would have a greater influence on people with a higher curvature utility function.

Formally, suppose $x_t$ is the earlier reward at time *t* and $x_{t+k}$ is a later larger reward at time $t + k$. We assume a time-separable CRRA utility function with a daily discounting factor $\delta$ and curvature of utility $0 < \alpha \le 1$ (for example, see,^10^), and additionally the present biased parameter $\beta$ if the earlier reward is immediate. Thus,

1 $$\begin{aligned} U(x_t, x_{t+K}) = \frac{1}{\alpha } \delta ^{t} x_t^{\alpha } + \frac{1}{\alpha } \beta I_{t} \delta ^{t+k} x^{\alpha }_{t+k} + \frac{1}{\alpha } (1 - I_{t}) \delta ^{t+k} x^{\alpha }_{t+k} \end{aligned}$$

where $I_t$ is an indicator variable which takes the value $I_{t} = 1$ if $t=0$ and $I_t = 0$ if $t>0$. If $t=0$, then an individual would choose the larger later reward $x_{k}$ if and only if;

2 $$\begin{aligned} x_0^{\alpha } \le \beta \delta ^{k}x_{k}^{\alpha } \end{aligned}$$

Therefore, individuals with $\delta \ge \delta ^*$, will choose the later reward where $\delta ^* = [\frac{1}{\beta }(\frac{x_0}{x_{k}})^{\alpha }]^{\frac{1}{K}}$ is the threshold.

In the cancellation treatment, we represent $x_0$ as $x_0 = y_0 + c$ and $x_{k}$ as $x_{k} = y _{k} + c$, where $c > 0$ is the common amount between $x_0$ and $x_{k}$. If individuals cancel the common amount, *c*, they would mentally represent $x_0$ as $y_0$ and $x_{k}$ as $y_{k}$. A person would choose $y_{k}$ over $y_{0}$ if and only if:

3 $$\begin{aligned} y_0^{\alpha } \le \beta \delta ^{k}y_{k}^{\alpha } \end{aligned}$$

Therefore, if people cancel out the common amount *c*, they will choose the later amount as long as their $\delta > \delta ^*_{c} = (\frac{y_0}{y_k})^{\frac{\alpha }{k}}$. As we show below, $\delta ^* > \delta ^*_{c}$.

4 $$\begin{aligned} \begin{aligned} x_{0}< x_{k} &\Rightarrow cx_{0} < cx_{k} \Rightarrow -cx_{0}>-cx_{k} \\& \Rightarrow x_{0}x_{k} - cx_{0}> x_{0}x_{k} -cx_{k} \\& \Rightarrow x_{0}(x_{k} - c)> x_{k}(x_{0} - c) \\& \Rightarrow \frac{x_{k} - c}{x_{0} -c}> \frac{x_{k}}{x_{0}} \\& \Rightarrow [\frac{1}{\beta }(\frac{x_0}{x_{k}})^{\alpha }]^{\frac{1}{K}}> [\frac{1}{\beta }(\frac{y_0}{y_{k}})^{\alpha }]^{\frac{1}{K}} \\& \Rightarrow \delta ^* > \delta ^*_{c} \end{aligned} \end{aligned}$$

The derivation also holds for $t > 0$, where $\beta =0$. Furthermore, the threshold decreases with an increase in $\alpha$, as $\delta ' (\alpha ) = \frac{\delta }{k}ln(z) < 0$ where $z=\frac{x_t}{x_{t+k}} < 1$. Therefore, we would expect an increase in people’s likelihood of choosing the later amount irrespective of the experimental condition as $\alpha$ increases. But note that as $\delta ^*_c < \delta ^*$, then $\delta ^{'*}_c< \delta ^{'*} < 0$. Therefore, $|\delta ^{'*}_c| > |\delta ^{'*}|$. Hence, the threshold to choose the later amount changes faster with a change in $\alpha$ when individuals cancel the common amounts. In other words, if individuals cancel out the common amount, their choices will be more sensitive to changes in $\alpha$ (or the curvature). Consistent with our theory, we find that those whose estimated utility curvature is high are more likely to choose the patient option under the cancellation condition.

Third, we investigate whether the cancellation heuristic, a decision-making bias in and of itself, can be used to counter a widespread shortcoming of individuals’ decision making-excessive intertemporal impatience. Using a survey study, we tested a policy application of the cancellation heuristic by assessing whether separating an amount from Social Security payments across different retirement ages would make people more likely to choose larger later Social Security payments. We find a substantial increase in patience when choices are represented with common payoff separated.

This research was approved by the Institutional Review Board of Nanyang Technological University. All experiments were conducted adhering to the guidelines and regulations laid down by the Institutional Review Board of Nanyang Technological University. Informed consent was obtained from all participants. No participants were dropped from the analyses in any experiment. All conditions and intertemporal choice measures are reported. In each experiment, data was analyzed only after the target sample size was met. All statistical analyses were done using Stata (StataCorp LLC, College Station, TX).

## Experiment 1

The goal of Experiment 1 was to provide an initial test of the cancellation heuristic in intertemporal choice in a field setting.

### Method

A research assistant approached people seated individually at a large cafeteria at Nanyang Technological University. Participants were asked whether they would complete a short survey in return for a gift coupon. Once participants signed the consent form, they were handed a tablet with the survey launched. To ensure a cover story for paying participants, we asked them to complete a filler survey. They were asked five questions about the cafeteria where they were seated (e.g., about its cleanliness, service, food quality), followed by five demographic questions. Thereafter, participants in the control condition were asked to choose between a $2 cafeteria voucher valid starting today and a $3 cafeteria voucher valid starting one week later, which would be valid at all stalls in the cafeteria where they were currently seated. Participants in the cancellation condition were asked to choose between a $1 cafeteria voucher valid starting today and a $2 cafeteria voucher valid for use one week later. They were also informed that they would receive an additional $1 voucher valid for the same period as the voucher of their choice.

Based on participants’ choices, the tablet then displayed an instruction for the research assistant to pay participants either vouchers worth $2 valid starting that day or vouchers worth $3 valid starting one week later. The appropriate validity start date was stamped on the vouchers. This methodology ensured identical transaction costs across both conditions because all participants immediately received the vouchers with the respective validity dates.

To determine the required sample size for Experiment 1, which was run in the cafeteria, we conducted a pilot study with 204 participants recruited on Amazon Mechanical Turk. The Amazon Mechanical Turk participants were randomly assigned to either the control or the cancellation condition. In the control condition, participants were asked to choose between $2 reward today versus a $3 reward in a week; 37% chose the later reward. However, this proportion increased to 63% in the cancellation condition, in which the choices were presented as $1 plus a $1 reward today versus a $2 plus a $1 reward in one week. A power analysis based on effect size $$w = .23$$, $$\alpha = .05$$, $$power = 80{\%}$$, $$df = 1$$, indicated that we would need to recruit a total of 147 participants. Thus, we collected data from 147 participants (72 women, 75 men; mean age 22.0 years) in the cafeteria.

### Results

A Chi-square analysis found that participants in the cancellation condition $(M = 77.8{\%}$, 95% CI = $[66.9{\%}, 85.8{\%}])$ were more likely to choose the larger later reward than those in the control condition $(M = 52.0{\%}$, 95% CI = $[40.9{\%}, 62.9{\%}])$, $\chi ^2(df = 1, N = 147) = 10.68$, $p = .001$, $w = .27$. A Fisher-exact confirmed this observation, $(p=0.002)$. Experiment 1 thus demonstrated that framing intertemporal options in a manner that allowed people to cancel out a seemingly identical component nudged people to choose the larger later option. Indeed, the proportion of participants choosing the larger later reward increased by a factor of 50% in the cancellation condition compared to the control condition.

## Experiment 2

### Method

Experiment 2’s goal was to provide further evidence of the cancellation heuristic using money as a reward, and importantly, to test whether people whose utility function has higher curvature would be more susceptible to the cancellation heuristic. We first measured the curvature of participants’ utility function by asking them to make a series of intertemporal choices. We then randomly assigned participants to either the control condition or the cancellation condition, and asked them to make a second series of intertemporal choices that were framed according to the condition to which they were assigned. A power analysis based on effect size $w = .27$ (from Experiment 1), $\alpha = .05$ (one-tailed), $power = 80{\%}$, $df = 1$, indicated that we would need to recruit a total of 85 participants. Compared to Experiment 1, Experiment 2 had multiple trials and hence greater statistical power. We posted an advertisement seeking 75 students to participate in a paid lab study on the Nanyang Business School behavioral lab paid subject pool. In response, 75 participants completed the study (33 women, 42 men; mean age 22 years). All participants completed two tasks.

#### Task 1

The first task was identical across the two conditions. We used the convex budget set method^10^ to measure participants’ time preference parameters, including the curvature of their utility function. In each of 20 choices, participants had 50 tokens that they could allocate between an earlier and a later period. The earlier period was either $t = 0$ days or $t = 7$ days. The later period was either $k = 35$ or $k = 70$ days away from the earlier period. Each token allocated to the earlier period had a value $a_t$ and each token allocated to the later period had a value of $a_{t+k} > a_t$. We used 5 different pairs of $(a_t; a_{t+k}) = {(0.19, 0.20), (0.18, 0.20), (0.16, 0.20), (0.14, 0.20), (0.20, 0.25)}$.

#### Task 2

Thereafter, we randomly assigned participants to either the control condition or to the cancellation condition. In both conditions, we presented participants with 20 choices between an earlier smaller reward and a later larger reward. The choices varied in the amount and also in the time of fulfillment. We used 5 different earlier rewards with values of $3, $4, $5, $7, and $8. The later reward was either $1 or $2 more than the earlier reward. The initial time was either $$t = 0$$ days or $$t = 7$$ days, and the later time was 7 days away from the initial time. The two conditions differed in the framing of choice. In the control condition, we used the standard framing. For example, participants were offered $3 today or $4 in a week. In the cancellation condition, the same choice was framed as $3 + $0 today or $3 + $1 in a week. The list of choices used is presented in Table 1.

**Table 1:** Stimuli and results from Experiment 2 (gains).

Row #	Earlier amount (t = 0)	Later amount (t = 7)	Baseline (%)	Cancellation (%)	Fisher exact test (*p*-value)
1	3	4	65	94.29	0.002
2	4	5	67.5	91.43	0.02
3	5	6	67.5	94.29	0.004
4	7	8	67.5	94.29	0.004
5	8	9	65	88.57	0.03
6	3	5	85	94.29	0.27
7	4	6	85	97.14	0.11
8	5	7	87.5	97.14	0.21
9	7	9	85	94.29	0.27
10	8	10	85	94.29	0.27

Row #	Earlier amount (t = 7)	Later amount (t =14)	Baseline (%)	Cancellation (%)	Fisher exact test (*p*-value)
11	3	4	42.5	77.14	0.004
12	4	5	45	74.29	0.02
13	5	6	45	77.14	0.005
14	7	8	45	77.14	0.005
15	8	9	50	77.14	0.02
16	3	5	80	88.57	0.36
17	4	6	82.5	91.43	0.32
18	5	7	80	85.71	0.56
19	7	9	77.5	91.43	0.12
20	8	10	80	82.86	0.77

Percentages indicate the percent of participants choosing the larger later gains.

To avoid a wealth effect across choices, participants were paid for one randomly selected choice out of the total 40 choices that they made. To minimize differences in risk and transaction costs for receiving payment earlier versus later, all participants were paid directly to their bank account on the day they were supposed to receive the money through a nation-wide, costless, online banking system. Further, following past research procedure^10^, participants who would be paid at a later date were provided the senior author’s business card with the amount and payment date noted on the back of the card. They were also informed that as per the university’s IRB rules, the researchers were obligated to pay on time. Participants were asked to contact the professor if they did not receive payment on the given date.

### Results

We first report the effect of the experimental manipulation in Task 2. For the 10 choices in which the later reward was $1 more than the earlier reward, participants chose the later reward in 56% of the trials in the control condition, $$SD = 41.92$$, 95% CI [42.59, 69.41], but in the cancellation condition, this proportion increased to 84.57%, $$SD = 29.34$$, 95% CI [74.49, 94.65] (Mann–Whitney U-test, $$p = 0.001$$, Cohen’s $$d = - 0.78$$). Table 1 shows that for each trial, when the later reward was $1 more than the earlier reward (Row 1–5 and Row 11–15), participants were significantly more likely to choose the larger later reward in the cancellation condition. When the later reward was $2 more than the earlier reward (Row 6–10 and Row 16–20), a larger number of participants still chose the later rewards in the cancellation condition than in the control condition, but this difference was no longer statistically significant. This is likely because overall, participants in both conditions chose the later reward at a high rate, leading to a ceiling effect. For these 10 choices, participants chose the later reward in 82.75% of the trials the control condition, $$SD = 33.35$$, 95% CI [72.08, 93.42], and in 91.71% of the trials in the cancellation condition, $$SD = 21.07$$, 95% CI [84.48, 98.95] (Mann–Whitney U-test, $$p = 0.4644$$, Cohen’s $$d=-0.32$$). We also found that overall, when we averaged across all choices that each participant made, participants were more likely to choose the later larger reward in the cancellation condition than in the control condition: 69.37% of the participants chose the later larger reward in the control condition, $$SD = 33.49$$, 95% CI [58.66, 80.08], whereas 88.14% did so in the cancellation condition, $$SD = 24.1$$, 95% CI [79.86, 96.42]. This difference was significant (Mann–Whitney U-test, $$p = 0.003$$, Cohen’s $$d=-0.63$$).

Following the procedure from past research^10^, we used the convex budget set to calculate each individual’s time preference parameters: $\alpha _i$ captures the curvature of each individual’s utility function, $\delta _i$ captures time-discounting, and $\beta _i$ captures the extent of present-biased preferences. The lower the $\alpha _i$, the greater the curvature of the participant’s utility curve. Four participants have a negative $\alpha$ due to inconsistent choices in the CBT trials. After dropping these participants, the descriptive statistics of the three parameters are: $\alpha [Mean = 0.8861 , SD= 0.1255 ]$, $\beta [Mean= 0.9977 , SD= 0.1645]$ and $\delta = [Mean= 0.9988 , SD = 0.0068]$. Table 2 presents the average marginal effect from a logistic regression on the Task 2 trial-level data (clustered at the level of participants), with participants’ choice as the dependent variable (coded 1 if they chose the larger later amount, and 0 if they chose the smaller earlier amount). The predictor variables were experimental condition (control condition = 0, cancellation condition = 1); the participant’s three time-discounting parameters; $t_0$, which denotes whether in the given trial, the earlier date was immediate (coded 1) or a week away (coded 0); and interaction terms between condition and $t_0$, and between condition and $\alpha _i$. Our regression analysis excludes participants with a negative $\alpha$.

**Table 2:** Logit regression for Experiment 2 (gains): later choice as dependent variable.

	(1) Later choice	(2) Later choice	(3) Later choice
*Cancellation*	0.249*** (0.077)	0.250*** (0.061)	1.173*** (0.366)
$t_0$		0.154*** (0.021)	0.145*** (0.031)
$\alpha _i$		1.285*** (0.247)	1.895*** (0.432)
$\delta _i$		9.677 (9.834)	12.591 (9.156)
$\beta _i$		− 0.459 (0.357)	− 0.482 (0.357)
$CancelationXt_0$			0.030 (0.042)
$Cancellation X \alpha _i$			− 1.059*** (0.397)
lnsig2u	3.595***	4.297***	4.305***
	(0.345)	(0.368)	(0.359)
Observations	710	660	660
Clusters (Subjects)	71	66	66

Standard errors in parentheses.

* $$p<.10$$; ** $$p<.05$$; *** $$p<.01$$.

In specification I, we found a significant positive coefficient of *Cancellation*, replicating our earlier finding that the cancellation condition increased the likelihood of choosing the later option compared to the baseline. In specification II, we controlled for time preference parameters and $t_0$. We again found a significant positive coefficient of *Cancellation*. Moreover, we found a positive significant coefficient of $\alpha _i$, implying that those with lower diminishing marginal utility were more patient. We also found a positive and significant coefficient of $t_0$. This observation is at odds with the theory of present-biased preference, which suggests that people are more patient when choosing between two alternatives in the future compared to alternatives that involve a choice between now versus later. Lastly, in specification III, we additionally controlled for the interaction term between cancellation and $\alpha _i$ and the interaction term between cancellation and $t_0$. The coefficient on interaction term *Cancellation*X$\alpha _i$ captured the underlying mechanism through which the cancellation heuristic allows participants to make a more patient choice. We found the predicted negative interaction between $\alpha _i$ and *Cancellation*. This finding indicates that the higher the curvature of participants’ utility curve (or lower the value of $\alpha _{i}$), the more influenced they were by the experimental manipulation. In other words, the cancellation condition was more successful in nudging participants to choose the later amount if they have a more curved utility function (i.e., a greater rate of diminishing marginal utility).

Experiment 2 replicated the findings of Experiment 1 across a range of choices. Furthermore, it provided support for the underlying mechanism—the curvature of people’s utility curve. The greater the curvature, the more susceptible participants were to the experimental manipulation. This finding is consistent with the idea that the cancellation effect works by moving both the smaller earlier reward and the larger later reward to an earlier position in people’s utility curve. This shift makes a bigger difference for people whose utility function has greater curvature.

## Experiment 3

### Method

Experiments 1 and 2 demonstrated the cancellation bias in the domain of gains. The goal of experiment 3 was to test whether the cancellation bias is also evident in the domain of losses. Contrary to the predictions of expected utility theory, previous research has found that people tend to choose larger later losses over smaller earlier losses (for example, see.,^11,12^). However, people would be better off choosing smaller earlier losses.

We hypothesized that the cancellation heuristic would also operate in the loss domain and nudge people to make more rational decisions-when a common amount is separated from the smaller earlier and the larger later loss, people would be more likely to choose the smaller earlier loss. This is because if canceling a common amount from both losses moves the two amounts to an earlier position in people’s utility curve, given the curvature of people’s utility curve, the additional disutility from the larger later loss would appear magnified; therefore, people would prefer to take the smaller earlier loss in the cancellation condition. For example, consider the choice between losing $10 today versus losing $11 in a week. Many people might prefer to postpone the loss and choose to lose $11 in a week. However, suppose this choice is framed as one between losing $0 + $10 today versus losing $1 + $10 in a week. If people ignore the “− $10,” then the choice is equivalent to losing $0 today versus $1 in a week. Clearly, losing nothing today would appear more attractive in this case. Thus, we hypothesized that the cancellation frame could be used to nudge people to choose smaller earlier losses over larger later losses.

A power analysis based on *Cohen's* $d = -0.60$ (from Experiment 2), $\alpha = 0.05$ (two-tailed), and $power = 80{\%}$ indicated that we would need to recruit 45 participants for each condition. We posted an advertisement seeking 120 students to participate in a paid lab study on the Nanyang Business School behavioral lab paid subject pool. In response, 115 participants completed the study (56 women, 59 men; mean age 19.93 years).

At the beginning of the session, the experimenter gave $10 in cash to each participant. Participants were instructed that they would not be able to keep all of the money and would have to return some of the money to the experimenter, and that this amount they would have to return would depend on their choices. Participants were then randomly allocated to either the control condition or the cancellation condition. In both conditions, we presented participants with 20 choices between an earlier smaller loss and a later larger loss (see Table 3). As in Experiment 2, the choices varied in the loss amount and the time of repayment. We used 5 different larger later losses with values of $7, $6, $5, $3, and $2. The earlier loss was either $1 or $2 less than the later loss. Note that the choices for each trial in the loss domain are symmetric to the gain domain. For each trial, after paying the loss, the remaining money that the participant kept is equal to a gain trial. The difference is that the earlier choice yielded the higher payoff in the loss domain, while the later choice yielded the higher payoff in the gain domain. The timings of the smaller earlier and larger later losses were either $$t = 0$$ days and $$t = 7$$ days from today, respectively, or $$t = 7$$ days and $$t = 14$$ days from today, respectively. The two conditions differed in the framing of choices. In the control condition, we used the standard framing. For example, participants were asked to choose between giving up $3 today or giving up $4 in a week. In the cancellation condition, the same choice was framed as give up $3 + give up $0 today or give up $3 + give up $1 in a week.

**Table 3:** Stimuli and results from Experiment 3 (losses).

Row #	Earlier Amount (t = 0)	Later Amount (t = 7)	Baseline (%)	Cancellation (%)	Fisher exact Test (*p*-value)
1	6	7	20.37	11.54	0.29
2	5	6	12.96	5.77	0.32
3	4	5	11.11	5.77	0.48
4	2	3	5.56	1.92	0.61
5	1	2	9.26	3.85	0.43
6	5	7	7.41	1.92	0.36
7	4	6	5.56	0	0.24
8	3	5	3.70	0	0.49
9	1	3	0	0	–
10	0	2	1.85	1.92	1

Row #	Earlier Amount (t = 7)	Later Amount (t = 14)	Baseline (%)	Cancellation (%)	Fisher exact Test (*p*-value)
11	6	7	16.67	3.85	0.05
12	5	6	9.26	1.92	0.20
13	4	5	22.22	0	0.00
14	2	3	7.41	0	0.11
15	1	2	5.56	1.92	0.61
16	5	7	5.56	1.92	0.61
17	4	6	3.70	0	0.49
18	3	5	1.85	0	1
19	1	3	1.85	0	1
20	0	2	0	5.77	0.11

Percentages indicate the percent of participants choosing the larger later losses.

The experiment was fully incentivized. One of the 20 choices that participants made was randomly chosen at the end of the experiment to determine the amount of money the participants had to return to the experimenter. To avoid any difference in transaction costs between returning money between the earlier and the later loss, all participants had to return the money to the experimenter on the appropriate date through a nation-wide, costless, online banking system. Participants were sent a reminder email on the day they had to return the money. Although participants had an incentive to not return the money, this incentive was constant across both experimental conditions and across the timing of the loss (no money was returned in the lab, and all transactions took place through the online banking system). Only 3 out of the 115 participants did not return the money.

### Results

For the 10 choices in which the later loss was $1 more than the earlier loss, participants chose the later loss in 12.03% of the trials in the control condition, $$SD = 21.92$$, 95% CI [6.05, 18.02]. But in the cancellation condition, this proportion decreased to 3.65%, $$SD = 10.85$$, 95% CI [0.63, 6.67] (Mann–Whitney U-test, $$p = 0.01$$, *Cohen's* $$d = 0.48$$). Table 2 shows that for most trials, participants were less likely to choose the larger later loss in the cancellation condition than in the control condition. However only two of the trials were statistically significant. The effect sizes were smaller than what we had observed in the gain domain. We discuss in the next paragraph why this might be the case. For the 10 choices in which the later loss was $2 more than the earlier loss, participants chose the later loss in 3.14% of the trials in the control condition, $$SD = 9.87$$, 95% CI [0.45, 5.84]. In the cancellation condition, this proportion decreased to 1.15%, $$SD = 4.27$$, 95% CI $$[-0.03, 2.34]$$, though the difference is not significant (Mann–Whitney U-test, $$p = 0.35$$, *Cohen's* $$d = 0.26$$). We also find that overall, when we averaged across all choices that each participant made, participants were less likely to choose the later larger loss in the cancellation condition than in the control condition: 7.6% of the participants chose the later larger loss in the control condition, $$SD = 14.06$$, 95% CI [3.75, 11.43], whereas only 2.4% of the participants did so in the cancellation condition, $$SD = 5.81$$, 95% CI [0.78, 4.02]. This difference was significant (Mann–Whitney U-test, $$p = 0.06$$, *Cohen's* $$d=0.47$$).

Experiment 3 demonstrated that the cancellation heuristic also operates in the loss domain in intertemporal choice. When a common amount was separated from a smaller earlier and a larger later loss, participants were more likely to choose the earlier loss. This finding once again indicates that the cancellation heuristic works by moving the options to an earlier point in individuals’ utility curve: the same absolute difference between the two losses appears larger with smaller amounts (because of diminishing marginal utility), and so people are more likely to choose smaller earlier losses than larger later losses.

At the level of individual trials, we found significant differences by condition in only two trials. However, in all other trials except one, the effect is consistently in the predicted direction, even if non-significant. One explanation is that in Experiment 3 (losses), we have a floor-effect on many trials in the baseline condition, with a small proportion of participants choosing the larger later loss. Thus, there is less room for the cancellation treatment to further reduce the extent to which participants chose the larger later loss. In contrast, in Experiment 2 (gains), there is more room for the cancellation treatment to move participants’ choices toward larger later gains. A second explanation is that due to loss aversion, people value a gain of a specific amount less than an equivalent loss^11,13^. This would imply that people’s utility function has a higher curvature in the gain domain than in the loss domain. Indeed, this is a core assumption of prospect theory^13^. As the cancellation effect is stronger at higher curvatures of the utility function, we would expect a stronger cancellation effect with gains than with losses.

## Experiment 4

In many situations, choosing larger later rewards in intertemporal choices can lead to an overall increase in individuals’ welfare. For example, the US Social Security Administration (SSA) allows people to start receiving retirement benefits between the age of 62 and 70, with the monthly lifetime payment increasing with each additional year delayed. Given the substantial increases in lifespan over the past half a century^14^ and the fact that Social Security payments have become most Americans’ sole source of retirement income^15^, claiming larger Social Security benefits starting at a later age would minimize longevity risk (i.e., the risk of having insufficient savings and retirement income in old age;^16^). Indeed, one of the suggestions of the US National Commission on Fiscal Responsibility and Reform was to use behavioral science knowledge to encourage US residents to delay the age at which they begin receiving retirement benefits^16,17^. We suggest that policymakers can use the cancellation heuristic to nudge people to delay the start of their retirement benefits.

In the present survey study, we tested whether the cancellation heuristic can help delay people’s choice of when to start receiving their Social Security benefits. We split social security payments into two virtually identical components—social security benefits and retirement supplement. We made the social security benefits equivalent to the benefits the person would be entitled to at the age of 62, and held this constant irrespective of the age at which the person retires. We made the retirement supplement equivalent to the entitled benefits at ages 63 to 70 minus their entitled benefits at age 62. Such a design would be similar to separating the entire smaller earlier reward from the larger later reward. We tested whether separating the benefits at age 62 from the benefits at ages 63 to 70 would increase participants’ likelihood of claiming retirement benefits at a later age.

A notable aspect of the present study is that it involves a choice among nine options, whereas all previous studies asked participants to choose between two options at a time. Thus, the present study would test whether the cancellation effect generalizes to complex choices that involve more than two options.

### Method

A power analysis based on the effect size $d = .48$ (the average effect size from two pilot studies), $\alpha = .05$ (two-tailed), $power = 80{\%}$, $df = 1$, indicated that we would need 140 participants. To ensure high power, we decided on a larger target sample size of 200 participants. A survey seeking 200 US residents was posted on MTurk; 215 participants (106 women, 94 men, 15 unreported; mean age 34.79 years) completed the survey. Participants were randomly assigned to either the control condition or the cancellation condition.

We first asked all participants, “Imagine that you just had your 62nd birthday.” After that, those in the control condition were instructed: “You are eligible to receive Social Security Benefits. Although your “Full Retirement Age” according to the Social Security Administration is 67 years, you can choose to start receiving your Social Security Benefits as early as age 62 or as late as age 70. You are eligible to receive $9,400 in Social Security Benefits at age 62. You can also postpone and start receiving your benefits at a later age. The later you start your benefits, the bigger your yearly Social Security Benefits. When would you like to start receiving your benefits?”

Those in the cancellation condition were instructed: “You are eligible to receive Social Security Benefits and a Retirement Supplement from the Social Security Administration. Although your “Full Retirement Age” according to the Social Security Administration is 67 years, you can choose to start receiving your Social Security Benefits and Retirement Supplement as early as age 62 or as late as age 70. You are eligible to receive $9400 in Social Security Benefits and Retirement Supplement starting at age 62. You can also postpone and start receiving your benefits at a later age. The later you start your benefits, the bigger your yearly Retirement Supplement. When would you like to start receiving your benefits?” Participants were presented with options outlined in Table 4. We predicted that in the cancellation condition, if people mentally cancel out the common $9400 social security benefits across all ages, they would be deciding between receiving $0 at age 62 and up to $7100 at age 70. The difference between the smaller earlier retirement income and the larger later retirement income would thus loom larger, leading participants to choose a larger later retirement benefit.

**Table 4:** Stimuli and results from Experiment 4. Percentages indicate the percent of participants under 62 years of age choosing each retirement age.

Age	Baseline		Cancellation		
	Social security benefit	% Choosing option	Social security benefit	Retirement supplement	% Choosing option
62	$9400	16	$9400	$0	6
63	$10,000	5	$9400	$600	6
64	$10,700	4	$9400	$1300	4
65	$11,600	16	$9400	$2200	14
66	$12,500	19	$9400	$3100	11
67	$13,400	12	$9400	$4000	18
68	$14,400	7	$9400	$5000	7
69	$15,500	1	$9400	$6100	2
70	$16,600	20	$9400	$7100	32

### Results

As the modal participant chose to receive social security benefits at age 70, an end-point of the response scale, we analyzed the data using the nonparametric Mann–Whitney U test. We found that participants in the cancellation condition, $(M = 66.89$ years, $SD = 2.65$, 95% CI [66.39, 67.40]), preferred to retire at a later age than those in the control condition, $(M = 66.05$ years, $SD = 2.66$, 95% CI [65.53, 66.55]), $z = 2.30$, $p = .02$, *Cohen's* $d =.32$. As we had 21 participants in our sample who were 62 years or older, and thus were already eligible to make their Social Security decision, we conducted an additional analysis while excluding these participants. Once again, we found that participants in the cancellation condition $(M = 67.02$ years, $SD = 2.57$, 95% CI [66.51, 67.53]), preferred to retire at a later age than those in the control condition, $(M = 66.05$ years, $SD = 2.67$, 95% CI [65.52, 66.61]), $z = 2.50$, $p = 0.01$, *Cohen's* $d = .37$. Table 4 presents the percentage of participants under 62 years of age choosing each option in each condition.

We additionally tested whether participants’ age and income moderated the effect of the cancellation treatment. We ran an OLS regression with the age for receiving social security benefits as the dependent variable. The predictor variables were a dummy variable for the cancellation condition, participants’ age, participants’ annual household income (measured in blocks of $10,000 starting from $0–$10,000 to $100,000), and the interaction of the cancellation dummy with participants’ age and income. We found that the cancellation variable was significant and positive, as predicted, but age $$(p = 0.684)$$, income $$(p = 0.808)$$, and their interactions with cancellation, *AgeXCancellation* $$(p = 0.460)$$ and *IncomeXCancellation* $$(p = 0.359)$$ were non-significant. Thus, the cancellation nudge was similarly effective for participants across different age- and income-brackets.

Experiment 4 demonstrated that the cancellation heuristic in intertemporal decision making can have policy implications: When Social Security payments were separated into two components, one that is constant and one that varies across different retirement ages, participants who had not yet reached the minimum retirement age were willing to delay their retirement by a full year. The effect size of the cancellation heuristic in delaying Social Security benefits is larger than that found in previous successful interventions to encourage people to delay their Social Security benefits, which has ranged from 4 months^18^ to 9 months^19^. Even though it is a survey study, this experiment provides the first evidence that cancellation heuristic may be used in real world settings and provides a choice architecture framework to nudge people to choose the later option using cancellation heuristic in the context of social security benefits. However, this study is a non-incentive-compatible survey study, so future research needs to examine whether its findings hold with incentivized choices.

## Discussion

The current research identified a novel choice architecture intervention to reduce people’s general tendency to be intertemporally impatient, that is, to choose smaller earlier rewards over larger later rewards, and to choose larger later losses over smaller earlier losses. This intervention drew upon the cancellation heuristic, the idea that people tend to mentally ignore or cancel out seemingly similar components of different options. The current research demonstrated that people spontaneously cancel out seemingly similar but factually non-identical components across different options. This general cancellation heuristic, when combined with a concave utility function, led to the prediction that when a common amount is separated from both the earlier and the later options, the same absolute difference between the two options will receive stronger psychological weight. Thus the costs of choosing the earlier option would become more obvious. Consistent with this idea, we found that people whose utility functions had greater curvature were more likely to switch from smaller earlier rewards to larger later rewards when a common amount was separated from both options.

The present research makes a significant contribution to the science of judgment and decision-making by arguing that the cancellation heuristic is more general than previously conceptualized. Past research on this topic exclusively focused on people’s tendency to ignore factually identical components across different options^7–9^. Indeed, past work on the isolation effect focused on very narrow cases in which ignoring common probabilities across various risky options switched one option from uncertainty to certainty^8^, or in which ignoring common time delays across various intertemporal options switched one option’s delivery date from a later date to today^9^. Other research has focused on the cancellation heuristic in which people ignore identical components of different risky options^7^. The current research demonstrates that people have a general tendency to ignore seemingly but not factually identical components across different options, and this cancellation heuristic influences people’s choices even in the absence of any discontinuities (e.g., without converting an uncertain option into a certain one, and without converting a later option to an immediate one).

In addition, the general form of the cancellation heuristic identified in the current research has significant policy implications. For example, our final experiment found that people can be nudged to choose larger later Social Security payments when the base payout at age 62 is separated from the payoffs at all ages (62-70). In this case, people seemed to ignore the common payout at age 62 and primarily focused on the additional payout that they would receive for each additional delay in their retirement age. This led people to choose to retire a full year later. Although this was a hypothetical choice, future research can use the intervention identified in the present research as one means of nudging people to delay their retirement age to minimize longevity risk.

In sum, the present research identified a novel element of choice architecture^4^ that has not received much attention-separating common elements of options. This strategy highlights the relative differences between the options, and thus can be used in contexts in which focusing on differences between options can nudge people to make more rational choices.

## Data Availability

The materials, data, and analyses for all studies are available at https://osf.io/7xn64/.
